# Ultraviolet-Irradiated All-Organic Nanocomposites with Polymer Dots for High-Temperature Capacitive Energy Storage

**DOI:** 10.1007/s40820-023-01230-2

**Published:** 2023-12-20

**Authors:** Jiale Ding, Yao Zhou, Wenhan Xu, Fan Yang, Danying Zhao, Yunhe Zhang, Zhenhua Jiang, Qing Wang

**Affiliations:** 1 College of Chemistry, Jilin University, Changchun, 130012 People’s Republic of China; 2 Department of Materials Science and Engineering, The Pennsylvania State University, University Park, PA 16802 USA; 3 Deutsches Elektronen-Synchrotron DESY, Notkestr. 85, 22607 Hamburg, Germany

**Keywords:** High-temperature energy storage, Polymer dots, Ultraviolet irradiation, All-organic composite dielectrics

## Abstract

Polymer dielectrics capable of operating efficiently at high electric fields and elevated temperatures are urgently demanded by next-generation electronics and electrical power systems. While inorganic fillers have been extensively utilized to improved high-temperature capacitive performance of dielectric polymers, the presence of thermodynamically incompatible organic and inorganic components may lead to concern about the long-term stability and also complicate film processing. Herein, zero-dimensional polymer dots with high electron affinity are introduced into photoactive allyl-containing poly(aryl ether sulfone) to form the all-organic polymer composites for high-temperature capacitive energy storage. Upon ultraviolet irradiation, the crosslinked polymer composites with polymer dots are efficient in suppressing electrical conduction at high electric fields and elevated temperatures, which significantly reduces the high-field energy loss of the composites at 200 °C. Accordingly, the ultraviolet-irradiated composite film exhibits a discharged energy density of 4.2 J cm^−3^ at 200 °C. Along with outstanding cyclic stability of capacitive performance at 200 °C, this work provides a promising class of dielectric materials for robust high-performance all-organic dielectric nanocomposites.

**Supplementary Information:**

The online version contains supplementary material available at 10.1007/s40820-023-01230-2.

## Highlights

- All-organic polymer composites for high-temperature capacitive energy storage.
- Zero-dimensional polymer dots with high electron affinity are used as fillers.
- Deep charge traps from UV-irradiated films reduce the high-field conduction loss.

**Supplementary Information:**

The online version contains supplementary material available at 10.1007/s40820-023-01230-2.

## Graphical abstract

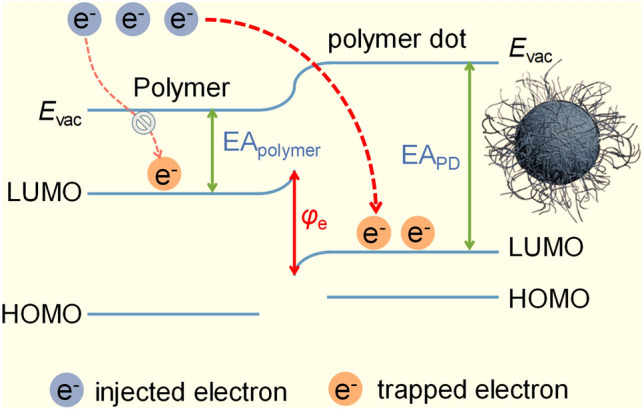

## Introduction

Electrostatic capacitors with the fastest charge–discharge rates and the highest power densities among the electrical energy storage devices are essential for advanced pulsed power systems and electrical propulsions [1–5]. Polymers are preferred dielectrics for high-energy–density capacitors because of their inherent advantages including high breakdown strength, low energy loss, great reliability and facile processability [6–11]. The emerging applications under extreme environments, such as transportation electrification and aerospace systems, require dielectric polymers capable of operating efficiently at high temperatures and high electric fields [12–17]. However, high-temperature capacitive performance of dielectric polymers is severely limited by exponentially increased leakage current with the applied field and temperature, which gives rise to sharp decreases in the electrical breakdown strength (*E*_*b*_), discharged energy density (*U*_*e*_) and charge–discharge efficiency (*η*) [3] For example, the maximum *U*_*e*_ of polyetherimide (PEI) precipitously drops from 5.2 J cm^−3^ at room temperature to 1.4 J cm^−3^ at 150 °C and 0.7 J cm^−3^ at 200 °C [13].

A variety of innovative approaches, including the layered structures [12, 18–20], the crosslinked polymers [1, 21, 22], the large-bandgap polymers and the polymer filled with molecular semiconductors [13, 23], have been developed to enhance the capacitive performance of dielectric polymers at elevated temperatures. The most popular method is the introduction of inorganic nanofillers with wide bandgaps (e.g., boron nitride [1, 24], alumina [25, 26], silica [27]) to form dielectric organic–inorganic polymer composites. The wide bandgap inorganic fillers have been demonstrated to be highly effective in reducing the charge injection from the electrodes and suppressing the electron transport within polymer matrix, which significantly improve the *U*_*e*_ and *η* of the dielectric composites at high temperatures. For example, the PEI composite filled with the core–shell structured nanoparticles composed of ZrO_2_ core and Al_2_O_3_ shell delivers a high *U*_*e*_ of 5.19 J cm^−3^ with a *η* of > 80% at 150 °C, benefiting from its several orders of magnitude lower leakage current density than that of PEI [28]. On the other hand, inorganic fillers are thermodynamically incompatible with organic polymers, which may lead to concern about the long-term stability of the resulting organic–inorganic composites, especially under elevated temperatures and high applied electric fields. Moreover, the large difference in the processability of inorganic fillers and organic polymers not only complicates the film processing but also could pose significant challenges for the large-scale production of uniform thin films with a few micrometer thickness [3].

Here, we present that all-organic dielectric polymer composites comprising high-electron-affinity polymer dots to suppress high-field electrical conduction and improve the *U*_*e*_ and *η* at high temperatures. Polymer dots (PDs), which are zero-dimensional polymer nanoparticles with diameters less than 10 nm [29, 30], exhibit similar viscoelastic and chemical properties to those of polymer matrix, making them particularly attractive to be utilized as organic nanofillers in all-organic composites. PDs consisting of a small ordered core and a relatively large surface composed of hyperbranched polymer segments would ensure their compatibility with polymers and maintain the processability of dielectric polymers. Moreover, the energy band structure of PDs can be adjusted to increase the electron affinity (i.e., the difference between the vacuum level and the lowest unoccupied molecular orbital (LUMO) level), thereby endowing them with high electron affinity (> 3.0 eV), which can be used to effectively capture the injected and excited electrons via strong electrostatic attraction. The large difference in electron affinity between the PDs and polymer matrix creates deep-energy-level charge traps to prevent the trapped electrons from escaping, thereby further reducing the leakage current. In particular, by ultraviolet (UV) irradiation of the photosensitive polymer composite filled with PDs, the crosslinking structure will be constructed without introducing excess impurities and the energy traps can be deepened due to the energy level change of PDs, which gives the composite a high *U*_*e*_ of 4.2 J cm^−3^ at 200 °C.

## Experimental Section

### Materials

*N*,*N*-Dimethylformamide (DMF, 99.9%) was dried with CaH_2_ and distilled before use. *N*,*N*-Dimethylacetamide (DMAc, 99.9%), citric acid (CA), ethylenediamine (EDA), K_2_CO_3_, and toluene were used without any further purification. Diallyl bisphenol A (DBA, analytical reagent grade) was obtained. 4,4′-difluorodiphenylsulfone (DPS, analytical reagent grade) from Energy Chemical was recrystallized from hot ethanol and dried in vacuum at 80 °C for 12 h before use.

### Preparation and UV Modification of All-Organic Composite Dielectrics

The all-organic composite films were prepared by solution casting. According to the desired concentration, 1 mg of polymer dot was dissolved in 3 mL DMAc. Meanwhile, 0.2 g of polymer was dissolved in 3 mL of DMAc and stirred for 5 h. Subsequently, the solutions of polymer dot and polymer matrix were mixed and stirred for 5 h. After that, the solution was dropped onto a clean glass slide (8 cm × 8 cm) and kept in an oven at 80 °C for 12 h to remove the solvent, and then heated to 100 and 120 °C for 2 h to remove the residual solvent. After cooling to room temperature, the film was placed in deionized water to peel off from the glass, and then dried in vacuum at 120 °C for 12 h. Ultimately, thin films with a thickness of 12–16 μm were obtained. Besides, the pure polymer films were obtained by the same method. The prepared thin films were irradiated in an UV chamber (UV intensity of 8 W and wavelength of 365 nm) for 40 min on each side to obtain the crosslinked thin films.

## Results and Discussion

### Design Principle and Structural Characterizations

**Fig. 1:**
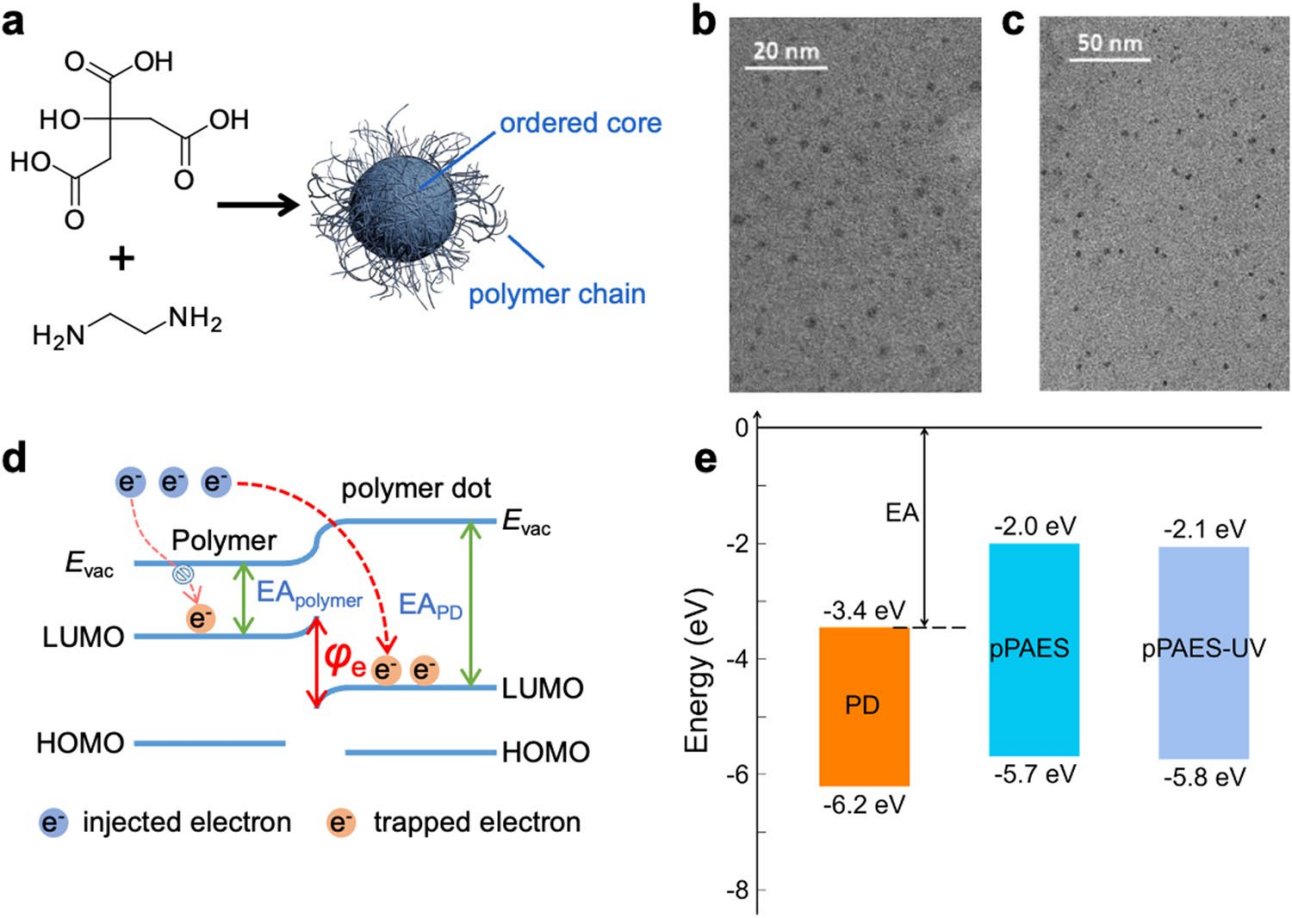
Design and structure of the all-organic polymer composite with PDs. **a** Schematic of the structure of the PD. **b** High-resolution TEM image of the PDs. **c** High-resolution TEM image of pPAES/PD with 0.5 wt% PDs. **d** Schematic of the charge trap introduced by the PDs. (*E*_vac_, vacuum level; *EA*_polymer_, electron affinity of the polymer; *EA*_PD_, electron affinity of the PDs; *φ*_e_, trap energy level). **e** Energy band diagrams of the PDs and pPAES

Allyl-containing poly(aryl ether sulfone) (pPAES) with relatively high glass transition temperature is chosen as the polymer matrix (Fig. S1), and more importantly, its photosensitive side group, allyl group, enables it to undergo cross-linking reaction under ultraviolet irradiation. The PDs are synthesized by a classical hydrothermal method using citric acid and ethylenediamine, as illustrated in Fig. 1a. The PDs with a diameter of about 4 nm (Fig. 1b) are found to be uniformly dispersed in pPAES matrix as revealed by transmission electron microscopy (TEM, Fig. 1c). The band structures of the PDs and pPAES obtained by UV absorption spectroscopy and cyclic voltammetry (Figs. S2 and S3) show that the electron affinity of the PDs (i.e., 3.4 eV) is significantly higher than that of pPAES (i.e., 2.0 eV), thus giving rise to a large trap energy level of 1.4 eV (Fig. 1d, e). Deep energy traps suppress the conductivity loss within the composite dielectric by capturing charges, thereby improving its energy storage performance ( Fig. S8). The optimal content of the PDs for improving the capacitive performance is determined to be 0.5 wt% as suggested by the highest *U*_*e*_ and *η* at 150 °C among the pPAES/PDs composites with various PD contents ( Fig. S4). Further, the presence of the unsaturated bonds (i.e., carbonyl and carbon–carbon double bond) in allyl groups in pPAES allows the formation of the crosslinking structures ( Fig. S5), which limits the movement and relaxation of molecular chains at high temperatures and further improves the thermal stability of pPAES and its all organic composites. Compared to thermal crosslinking, ultraviolet (UV) light-induced crosslinking offers the advantages of compatibility with the current film processing of dielectric polymers, processing flexibility, high efficiency and environmental friendliness. The crosslinking of pPAES and its all organic composites under UV irradiation at 365 nm (Fig. S6) is confirmed by the FTIR spectra and the gel content measurement (Fig. S7), and according to the thermal-loss curves (Fig. S8), the crosslinking structure effectively improves the thermal stability of pPAES and its all organic composites. Interestingly, we find that after UV irradiation, the functional groups (Fig. S9) and internal carbon nucleus conjugation (Fig. S10) of polymer dots also change due to their unsaturated flexible structures, which will lead to the formation of more deep traps and help to further improve the high-temperature energy storage of the dielectrics.

### Energy Traps and Leakage Currents

**Fig. 2:**
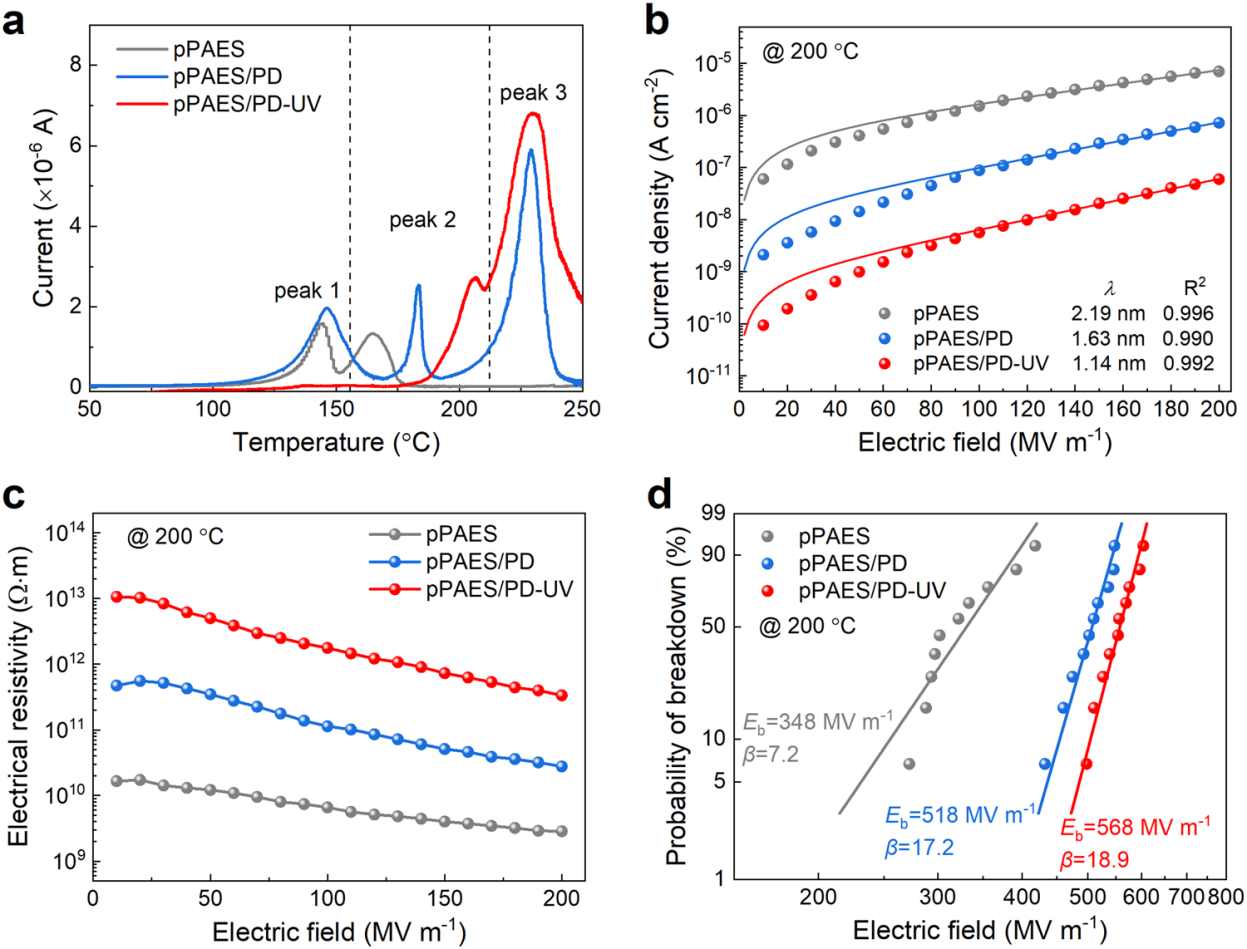
Electrical properties of the polymer composite with PDs. **a** Thermally stimulated depolarization currents of pPAES, pPAES/PD and pPAES/PD-UV. **b** Conduction current density as a function of electric field at 200 °C of pPAES-UV, pPAES/PD and pPAES/PD-UV (solid curves represent fit to hopping conduction mechanism). **c** Electric field dependent resistivity of pPAES, pPAES/PD and pPAES/PD-UV at 200 °C. **d** Two-parameter Weibull distribution analysis of the breakdown strength of pPAES, pPAES/PD and pPAES/PD-UV at 200 °C

The thermally stimulated depolarization current (TSDC) measurements prove the presence of deep traps with a trap level estimated to be around 1.5 eV (peak 3 at ~ 230 °C) in the pPAES/PDs composites as a result of the introduced high-electron-affinity PDs (Fig. 2a). In the TSDC spectra, peak 1 at ~ 140 °C arises from the local dipolar orientation of the pendant propenyl side groups in pPAES, while peak 2 corresponding to the glass transition of the polymer shifts to a higher temperature in pPAES/PDs because of the enhanced interaction between pPAES and the PDs. The crosslinking leads to the disappearance of peak 1 and shift of peak 2 to a higher temperature in the UV-irradiated film pPAES-UV (Fig. S11) and pPAES/PD-UV. Importantly, peak 3 in pPAES/PD-UV exhibits a largely increased peak area compared to that of pPAES/PD, indicating that deep traps can be formed in pPAES/PD-UV due to the changes in PDs after UV irradiation.

Consequently, as shown in Figs. S12 and 2b, the conduction current density of pPAES/PD-UV at 150 and 200 °C is one and two orders of magnitude lower than those of pPAES and pPAES/PD, respectively. The conduction current density increases exponentially with the applied field, suggestive of the hopping conduction mechanism in the polymers [31, 32]. According to the hopping conduction model, the conduction current density *J* is given as $J = 2ne\lambda \nu \times \exp \left( {\frac{{ - E_{a} }}{kT}} \right) \times \sinh \left( {\frac{ - \lambda eE}{{2kT}}} \right)$, where *n* is the charge carrier density, *λ* is the hopping distance, *ν* is the attempt-to-escape rate, *E*_*a*_ is the activation energy, *e* is the element charge, *k* is the Boltzmann constant, *T* is the absolute temperature and *E* is the applied electric field. The experimental data fit well with the equation with goodness of fit, *R*^*2*^, ranging from 0.990 to 0.996, further confirming that hopping conduction is the major conduction mechanism in the polymer composites at elevated temperatures and high electric fields. The hopping distance *λ* derived from the fitted curves decreases from 2.19 nm of pPAES to 1.63 nm of pPAES/PD and further to 1.14 nm of pPAES/PD-UV. The shorter hopping distance corresponds to a deeper trap depth and higher trap density, which are well consistent with the TSDC results.

As the result of suppressed conduction current, the introduction of PDs and crosslinking significantly enhance the electrical resistivity of the composites at 200 °C (Fig. 2c). The electrical resistivity of pPAES/PD-UV is increased by more than one and two orders of magnitude than pPAES/PD and pPAES at 200 °C, respectively. For example, at 200 °C and 200 MV m^−1^, the electrical resistivities of pPAES/PD-UV, pPAES/PD and pPAES are 3.4 × 10^11^, 2.8 × 10^10^, 2.9 × 10^9^ Ω m, respectively. The electrical resistivity of pPAES/PD-UV is also higher than the current polymer dielectrics, e.g., the electrical resistivity of PEI, the state-of-the-art high-temperature dielectric polymer, is 7.6 × 10^10^ Ω·m at 200 °C and 200 MV m^−1^. Consequently, pPAES/PD-UV has a *E*_*b*_ of 587 MV m^−1^ at 150 °C and 568 MV m^−1^ at 200 °C (Figs. S13 and 2d), which is significantly higher than that of pPAES (i.e., 416 MV m^−1^ at 150 °C and 348 MV m^−1^ at 200 °C) and also exceeds the existing dielectric polymers, e.g., *E*_*b*_ of PEI is only 484 MV m^−1^ at 200 °C. The evaluation of the weak-field dielectric constant (*K*) and dissipation factor (*DF*) shows that pPAES/PD-UV provides a *K* of 3.8 and a *DF* of 0.0037 (Fig. S14), representing a ~ 10% increase in *K* and ~ 35% reduction in *DF* compared to pPAES. Compared to pristine pPAES and pPAES/PD, the dielectric constants of cross-linked pPAES-UV and pPAES/PD-UV decrease from 3.5 and 4.0 to 3.2 and 3.8, respectively. pPAES-UV and pPAES/PD-UV exhibit stable dielectric properties in the temperature range of 20 to 200 °C, and the values of dissipation factors also decrease compared to those before UV irradiation.

### Energy Storage Performance

**Fig. 3:**
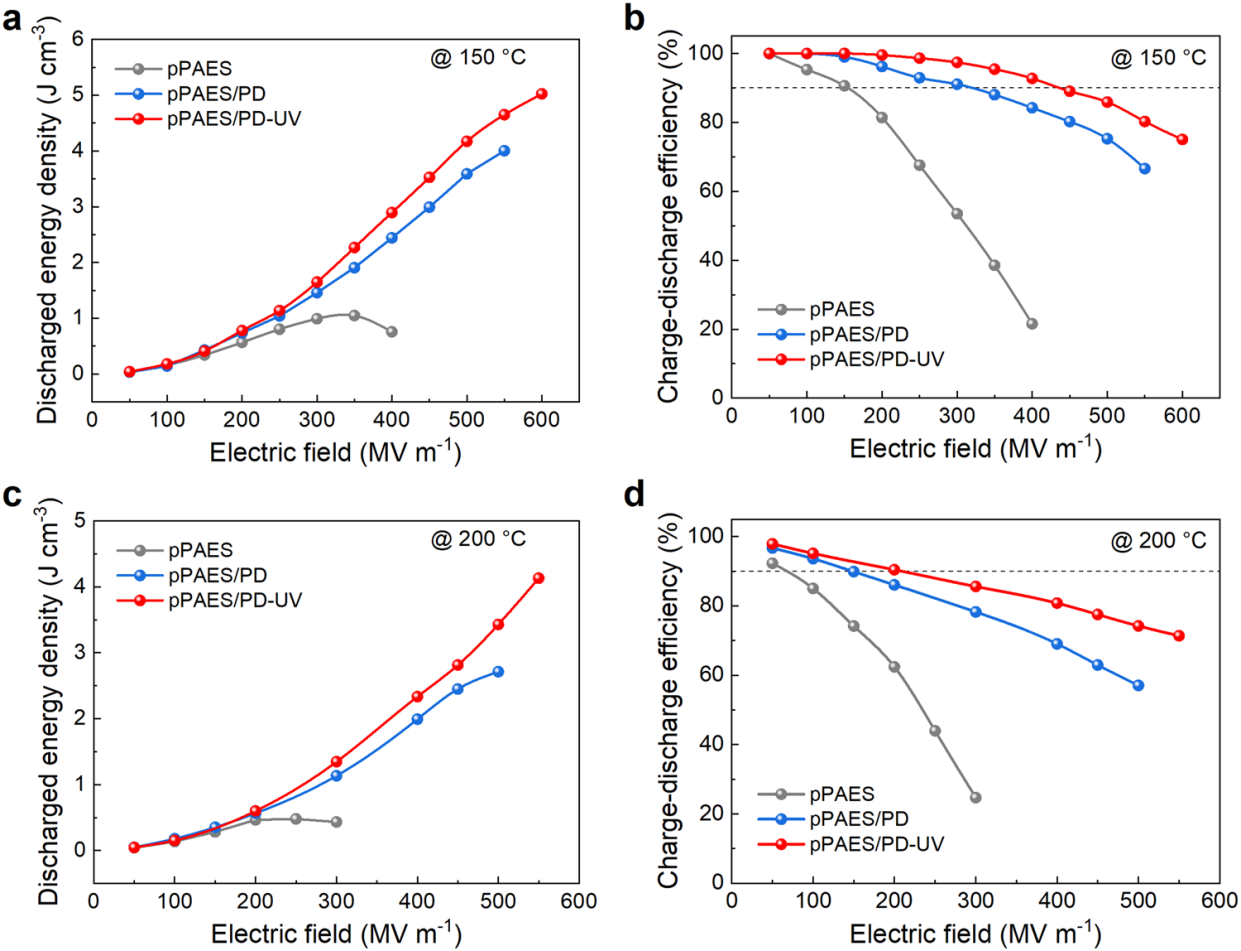
Capacitive energy storage performance of the polymer composite with PDs. **a** Discharged energy density and **b** charge–discharge efficiency of pPAES, pPAES/PD and pPAES/PD-UV at 150 °C. **c** Discharged energy density and **d** charge–discharge efficiency of pPAES, pPAES/PD and pPAES/PD-UV at 200 °C

Due to the suppressed conduction loss and increased *K*, the high-temperature capacitive performance of the all-organic pPAES/PD composite is drastically improved relative to pPAES. Figure 3 summarizes the capacitive energy storage performance derived from the unipolar electric displacement-electric field (*D–E*) loops ( Figs. S15 and S16) at 150 and 200 °C. At 150 °C and 400 MV m^−1^, pPAES/PD exhibits an *U*_*e*_ of 2.44 J cm^−3^ with a *η* of 84.2%, whereas the *U*_*e*_ and *η* of pPAES are only 0.76 J cm^−3^ and 21.6%, respectively. Furthermore, to illustrate the generality of the PDs, polyphenylsulfone (PPSU) is also employed as the polymer matrix. Interestingly, an improved *U*_*e*_ of 2.26 J cm^−3^ and *η* of 77.4% are found in the PPSU/PD composite with respect to the *U*_*e*_ of 1.03 J cm^−3^ and *η* of 36.9% of pristine PPSU at 150 °C (Figs. S17 and S18).

The crosslinking further enhances the capacitive performance, e.g., pPAES/PD-UV delivers an *U*_*e*_ of 5.0 J cm^−3^ at 150 °C, representing 25% and 400% increase compared to pPAES/PD and pPAES, respectively (Fig. 3a, b). As temperature increases to 200 °C, the improvement of the capacitive energy storage performance in pPAES/PD-UV is much more pronounced (Fig. 3c, d), i.e., *U*_*e*_ of 4.2 J cm^−3^ obtained in pPAES/PD-UV is 8 times that of pPAES. The *U*_*e*_ and *η* of the PPSU-based composites with the PDs have also been compared before and after UV irradiation (Figs. S17 and S18). Similarly, PPSU/PD-UV significantly outperforms PPSU/PD in both *U*_*e*_ and *η*, again demonstrating the effectiveness of the UV irradiation approach.

**Fig. 4:**
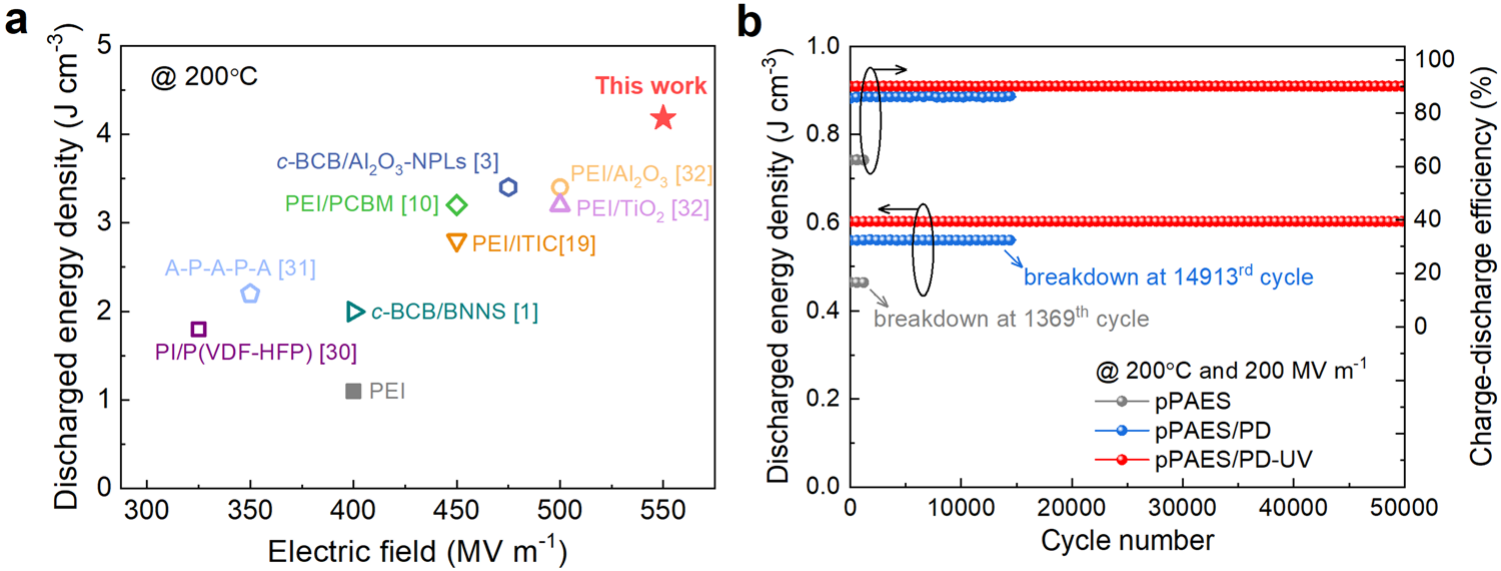
a Comparison of the maximum energy storage density of pPAES/PD-UV and other high-temperature dielectrics polymers and composites at 200 °C. **b** Cyclic performance of pPAES, pPAES/PD and pPAES/PD-UV at 200 °C and 200 MV m^−1^

We have compared the maximum *U*_e_ of pPAES/PD-UV to those of the current high-temperature dielectric polymers and composites at 200 °C (Fig. 4a) [1, 3, 13, 33–35]. Apparently, the *U*_*e*_ of 4.2 J cm^−3^ achieved in pPAES/PD-UV exceeds the existing polymers and polymer composites while with comparable efficiency, such as 3.4 J cm^−3^ of *c*-BCB/Al_2_O_3_ NPLs and 1.1 J cm^−3^ of PEI. Recently, a series of polyolefins [36] and their copolymers [37] have been developed to exhibit excellent high-temperature energy storage properties, e.g., a maximum discharge density of 8.37 J cm^−3^ at 200 °C. The *U*_e_ obtained in pPAES/PD-UV at 200 °C is comparable to the room temperature value of the state-of-the-art commercial polymer dielectrics, biaxially oriented polypropylene (BOPP) of ~ 4 J cm^−3^, paving the way for direct application of the all-organic polymer films under extreme temperatures without external cooling. The pPAES/PD-UV film also exhibit superior stability in the cyclic charge–discharge test under extreme conditions. At 200 MV m^−1^ and 200 °C, the *U*_*e*_ and *η* of the pPAES/PD-UV film are highly stable over 50,000 continuous charge–discharge cycles at 200 °C, while the pPAES and pPAES/PD films can only operate up to 1369 and 14,913 cycles, respectively (Fig. 4b).

## Conclusion

In summary, we demonstrate that the PDs with high electron affinity can be introduced into dielectric polymers to improve the high-temperature capacitive energy storage performance. The PDs offer a series of advantages, such as ease of synthesis, low cost, excellent compatibility and great stability. The TSDC results prove that the PDs function as deep traps to capture the injected and excited electrons, thus reducing the leakage current and improving the high-temperature capacitive energy storage performance. UV irradiation of the photosensitive polymer composite filled with PDs constructs the crosslinking structure and deepens charge traps to inhibit molecular chain relaxation and electron transport at elevated temperature. As a result, the maximum discharged energy density of the UV-irradiated all-organic composite achieved at 200 °C. Moreover, the energy density and efficiency of the UV-crosslinked all-organic composites show no sign of degradation after 50,000 charge–discharge cycles at 200 °C and 200 MV m^−1^. This work establishes PDs and UV irradiation as a promising material design platform to address the current challenges of scalable high-temperature dielectric polymers with robust and exceptional capacitive energy storage performance.

## Supplementary Information

Below is the link to the electronic supplementary material.

Supplementary file1 (PDF 1680 KB)
