# Supplementary material for: Ultraviolet-Irradiated All-Organic Nanocomposites with Polymer Dots for High-Temperature Capacitive Energy Storage
Source: Nanomicro Lett. 2023 Dec 20;16:59. doi: 10.1007/s40820-023-01230-2 (PMC10733267; doi:10.1007/s40820-023-01230-2)
Supplement: Supplementary file 1 — Supplementary file1 (PDF 1680 KB) [file 40820_2023_1230_MOESM1_ESM.pdf]

Nano-Micro Letters

Supporting Information For

Ultraviolet-Irradiated All-Organic Nanocomposites with Polymer Dots for High-Temperature Capacitive Energy Storage

Jiale Ding[1,†], Yao Zhou[2,†], Wenhan Xu[2,3,*], Fan Yang[1], Danying Zhao[1], Yunhe Zhang[1,*], Zhenhua Jiang[1], and Qing Wang[2,*]

[1] College of Chemistry, Jilin University, Changchun 130012, P. R. China

[2] Department of Materials Science and Engineering, Pennsylvania State University, University Park, PA 16802, USA

[3] Deutsches Elektronen-Synchrotron DESY, Notkestr. 85, 22607 Hamburg, Germany

†J. Ding and Y. Zhou contributed equally to this work.

*Corresponding authors. E-mail: wenhan.xu@desy.de (Wenhan Xu), zhangyunhe@jlu.edu.cn (Yunhe Zhang), wang@matse.psu.edu (Qing Wang)

S1 Experimental Section

S1.1 Preparation of Polymer Dots

The classic hydrothermal polymerization is used to prepare the polymer dot, and the specific process is as follows: citric acid (1.0507 g) and ethylenediamine (335 μL) were dissolved in deionized water (10 mL). The solution was then transferred to a poly(tetrafluoroethylene) Teflon- lined autoclave placed in an oven at 200 °C for 5 h. After the reaction was completed and cooled to room temperature, the mixed solution was transferred to a dialysis bag for dialysis and purification for 24 h, and then freeze-dried to obtain brown-black polymer dot. The prepared polymer dot was dispersed in DMF.

S1.2 Preparation of pPAES

Diallyl bisphenol A (12.337 g, 0.040 mol), 4,4′-difluorodiphenylsulfone (10.682 g, 0.040 mol), and K_2CO_3 (6.082 g, 0.044 mol) were dissolved in DMAc (82 mL) and toluene (26 mL), and the solution was stirred at 160 °C under N_2 for 7 h. After the reaction was completed and cool to room temperature, the product was poured into deionized water and filtered. The residue was washed with methanol and deionized water, and then dried under vacuum at 80 °C for 48 h.

S1.3 Characterization

Transmission electron microscopy (TEM) images of polymer dots were obtai ned by JEOL JEM-1200EX microscope at 160 kV. UV2501-PC spectrophotometer was used to get Ultraviolet- visible (UV-vis) absorption spectra. The electrochemical data were obtained from Bio-Logic SP- 150 electrochemical workstation. Fourier transform infrared spectroscopy (FTIR) was obtained by Varian Digilab FTS-8010 spectrometer with the mode of attenuated total reflectance and used ZnSe crystal as the contact to the samples. Typically, each spectrum was obtained by accumulating 32 scans with a resolution of 8 cm^{-1}. Dielectric constant and dissipation factors were measured using an Agilent 4294A LCR meter and EC1A oven. An improved Sawyer-Tower circuit was used to collect Electric displacement-electric field (*D*-*E*) loops, in

which the samples were subjected to triangular unipolar electric field with the frequency of 100 Hz. Dielectric breakdown strength was obtained by Trek Model 610C amplifier with the method of electrostatic pull-down and the DC voltage pressurization rate of 500 V s^{-1}. The breakdown tests were carried out in silicone oil bath to reduce surface corona discharge. Leakage current density was obtained using a Keithley Model 6517A electrometer equipped with a Trek Model 610C amplifier. Keithley Model 6517A electrometer was uesd to obtain thermally stimulated depolarization current (TSDC) according to the following steps. Firstly, the samples were heated to 250 °C and polarized at the electric field of 50 MV m^{-1} for 30 min, and then were cooled to -10 °C rapidly under this applied electric field. Afterward, the electric field was removed and the experimental system was changed to short-circuit mode and kept for 5 min. Finally, the samples were heated to 200 °C at the heating rate of 5 °C min^{-1} and the current during the heating process was collected by the experimental system. The trap energy level *ATSDC* can be obtained from the TSDC curves according to the equation,

$$A_{TSDC} = \frac{2.47 \times k_B \times T_p^{\,2}}{\Delta T}$$

where kB is the Boltzmann constant, Tp is the temperature corresponding to the peak current and ΔT is the peak width at half height. Copper electrodes with the thickness of 50 nm were deposited on both sides of the electrical test films.

S2 Supplementary Figures

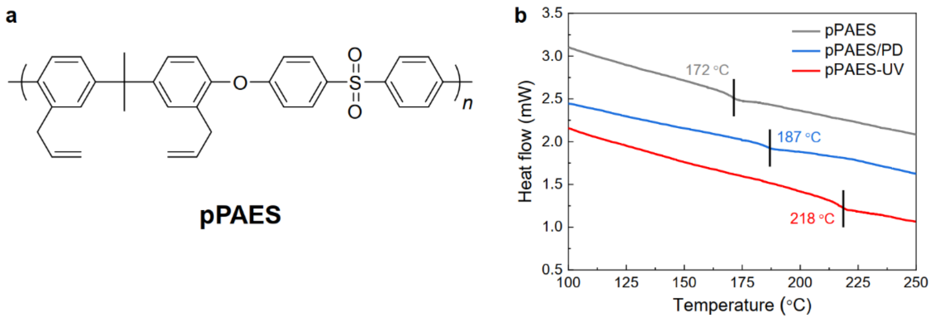

Fig. S1 (**a**) Chemical structure of pPAES. (**b**) DSC curves of pPAES, pPAES/PD and pPAES-UV

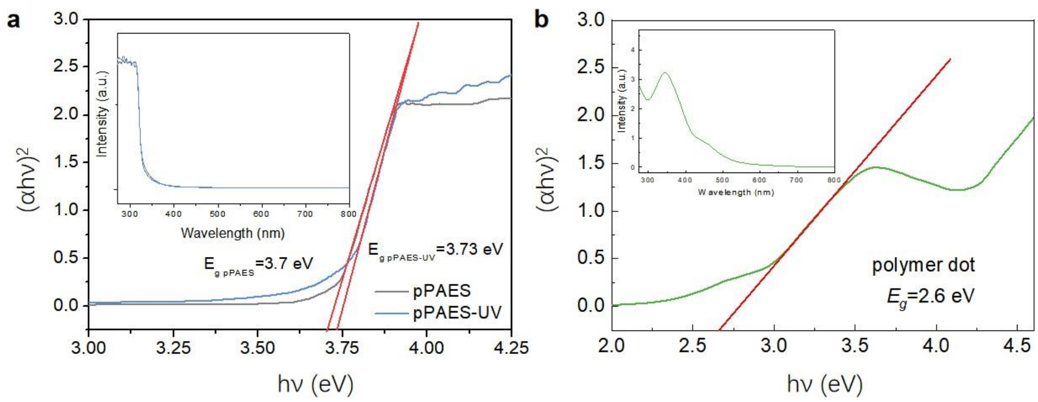

Fig. S2 UV-vis absorption spectra of (**a**) pPAES pPAES-UV, and (**b**) polymer dot in 0.05 mg mL^{-1} DMF solution

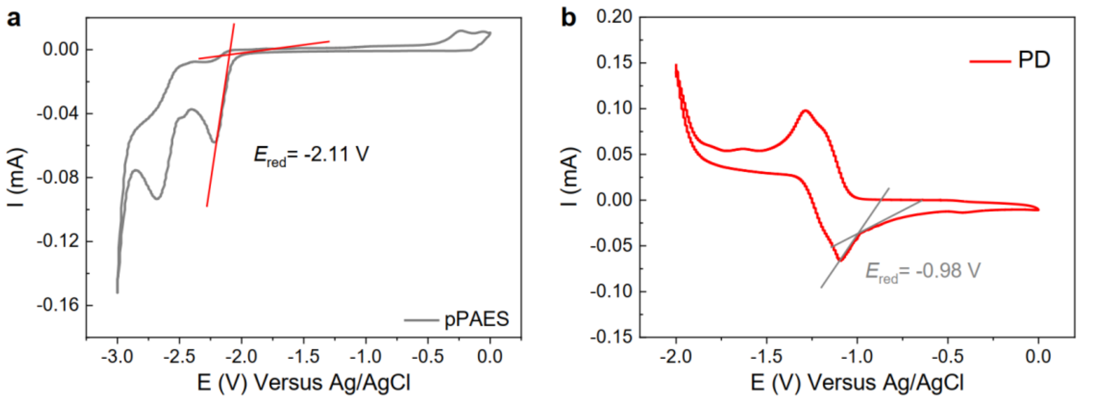

Fig. S3 Cyclic voltammogram of (**a**) pPAES (**b**) polymer dot in 0.1 mol L^{-1} $(Bu)_4NBF_4$ DMF solution

The HOMO and LUMO energy levels were calculated according to the following equations,

$$E_{LUMO} = E_{red} + 4.4$$

$$E_{HOMO} = E_{LUMO} + \Delta E$$

where E_{red} is the onset of reduction potential. ΔE is the bandgap calculated from the UV-vis spectra (e.g., ΔE_{PD} = 2.8 eV, ΔE_{pPAES} =3.7 eV, ΔE_{PPSU} =3.8 eV), and 4.4 eV corresponds to the potential of the reference electrode Ag/AgCl

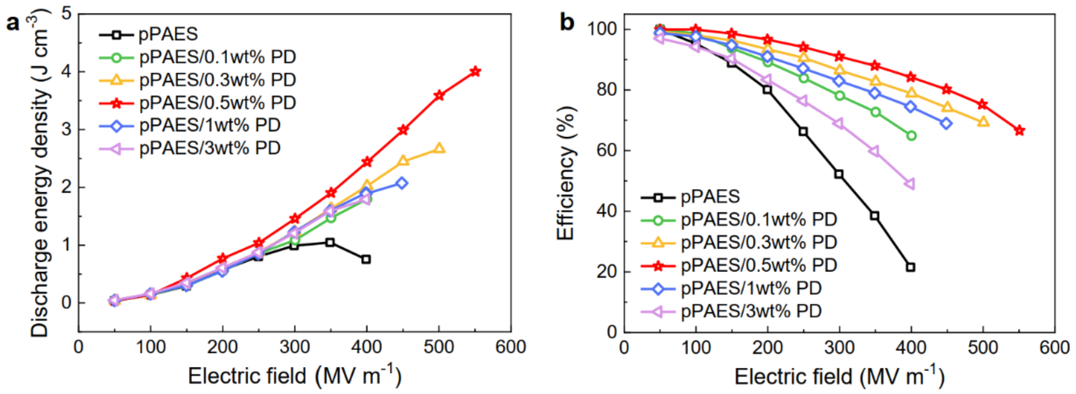

Fig. S4 (**a**) Discharged energy density and (**b**) charge-discharge efficiency of pPAES and pPAES/PD with different PD contents at 150 °C

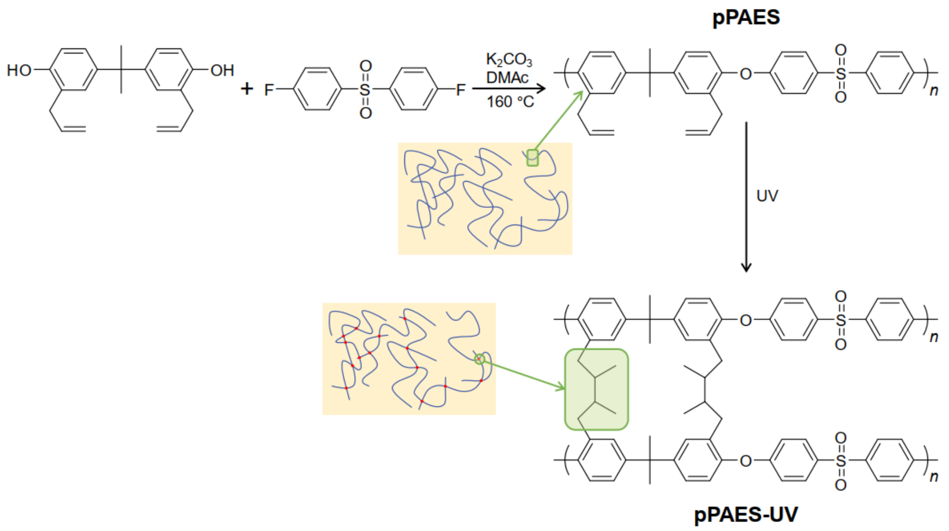

Fig. S5 Crosslinking reaction of pPAES under UV irradiation

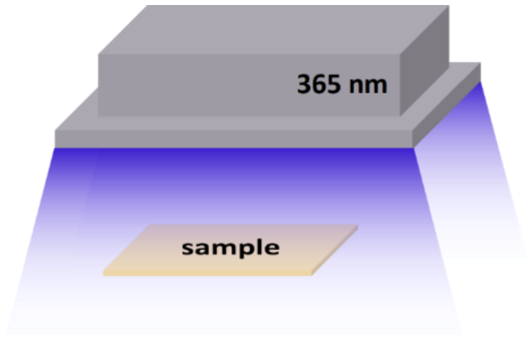

Fig. S6 Schematic of the UV irradiation

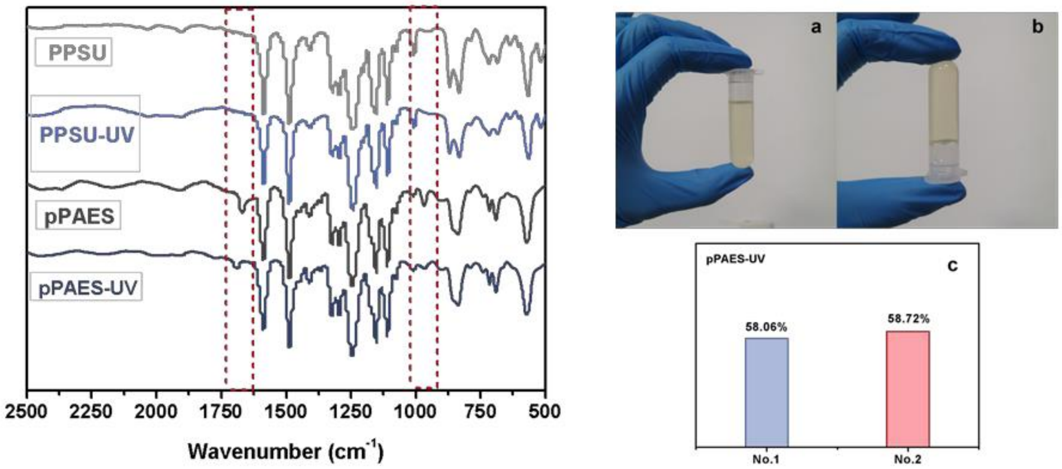

Fig. S7 (**a**) FTIR spectra of PPSU, PPSU-UV, pPAES and pPAES-UV. Images of pPAES in DMF solution (**b**) before and (**c**) after UV irradiation. (**d**) The gel content of different batches of crosslinked pPAES-UV

As shown in **Fig. S7a**, there is no change in the FTIR spectra of PPSU and PPSU-UV, indicating that there is no UV crosslinking reaction in PPSU. While in pPAES, because of the presence of photosensitive allyl groups, it can be observed that the absorption peaks of propylene side groups at 1668 and 967 cm^{-1} in pPAES-UV are obviously weakened, which indicates the occurrence of the crosslinking reaction. **Fig. S7b, c** shows the images of pPAES in DMF solution before and after UV irradiation. It can be seen that after UV irradiation, the original solution showed a non-flowing gel state. Moreover, the gel content test showed that the gel content of the pPAES-UV was about 58%.

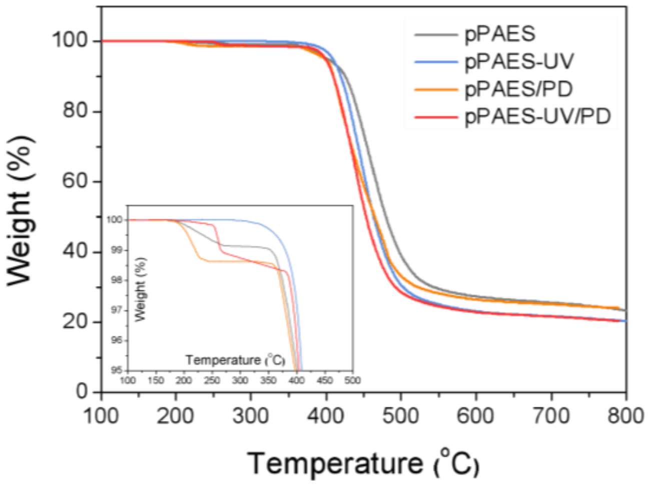

Fig. S8 TGA curves of pPAES, pPAES-UV, pPAES/PD and pPAES/PD-UV

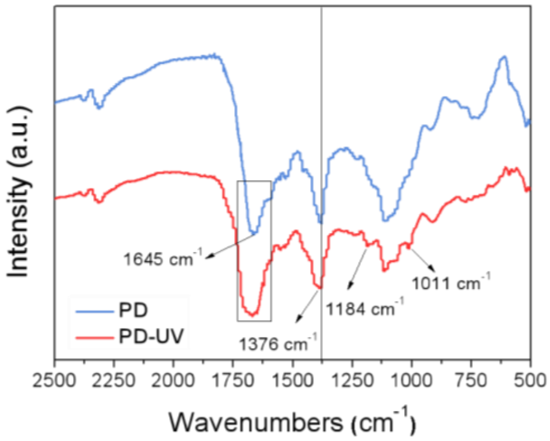

Fig. S9 Normalized FT-IR spectra of PD and PD-UV. The normalization is based on the stretching vibration peak of the C-N bond at 1230 cm^{-1}

As shown in the FT-IR spectra, the presence of C=O and C=C vibrational absorption peaks located in 1645 cm^{-1} in PD indicates the presence of unsaturated bonds. It is worth noting that after UV irradiation, the peak intensity of vibration absorption at 1645 cm^{-1} increases, and there are bending and stretching vibration peaks of C−H in alkene located at 1184 cm^{-1} and 1011 cm^{-1}, indicating the increase in the content of unsaturated carbon in PD.

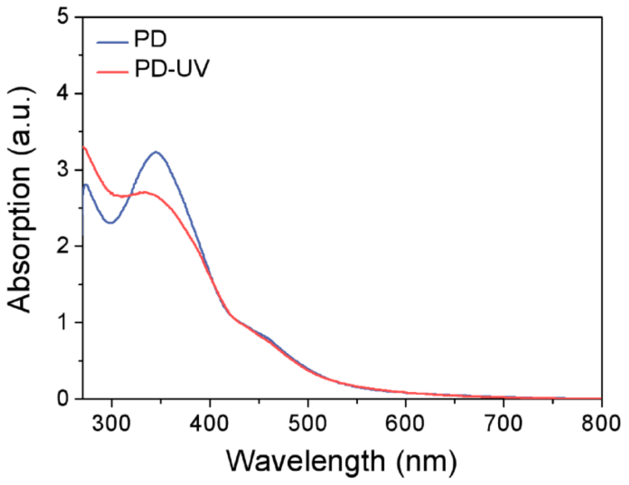

Fig. S10 UV-vis absorption spectra of PD and PD-UV in 0.05 mg mL^{-1} DMF solution

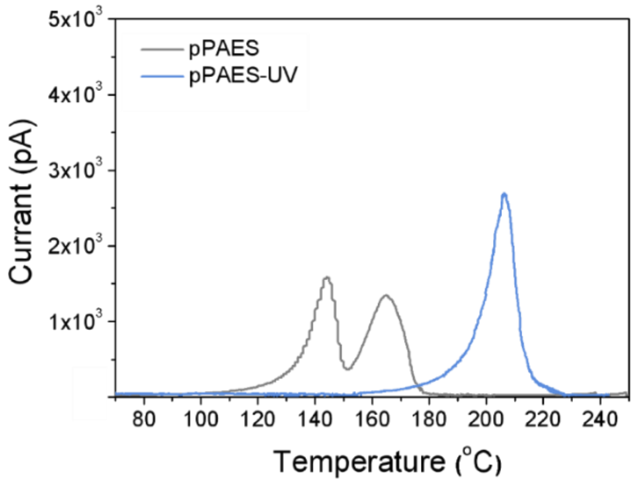

Fig. S11 Thermally stimulated depolarization currents of pPAES and pPAES-UV

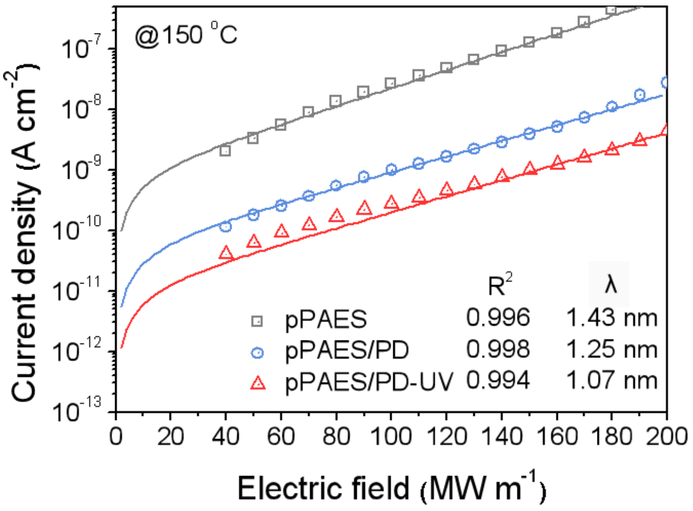

Fig. S12 Conduction current density as a function of electric field of pPAES, pPAES/PD and pPAES/PD-UV at 150 °C (solid curves represent fit to hopping conduction mechanism)

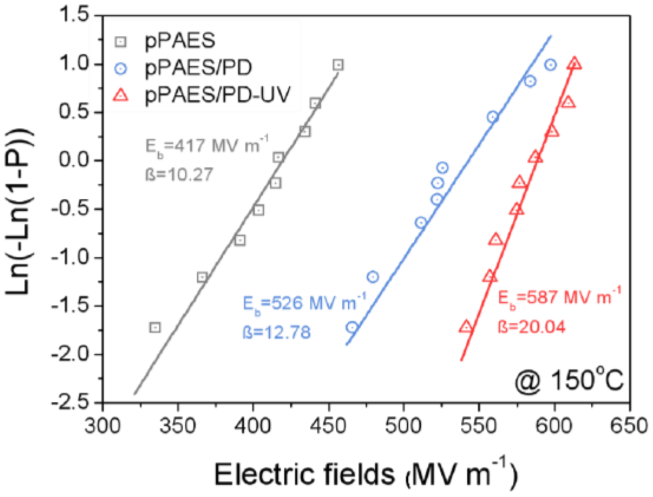

Fig. S13 Two-parameter Weibull distribution analysis of the breakdown strength of pPAES, pPAES/PD and pPAES/PD-UV at 150 °C

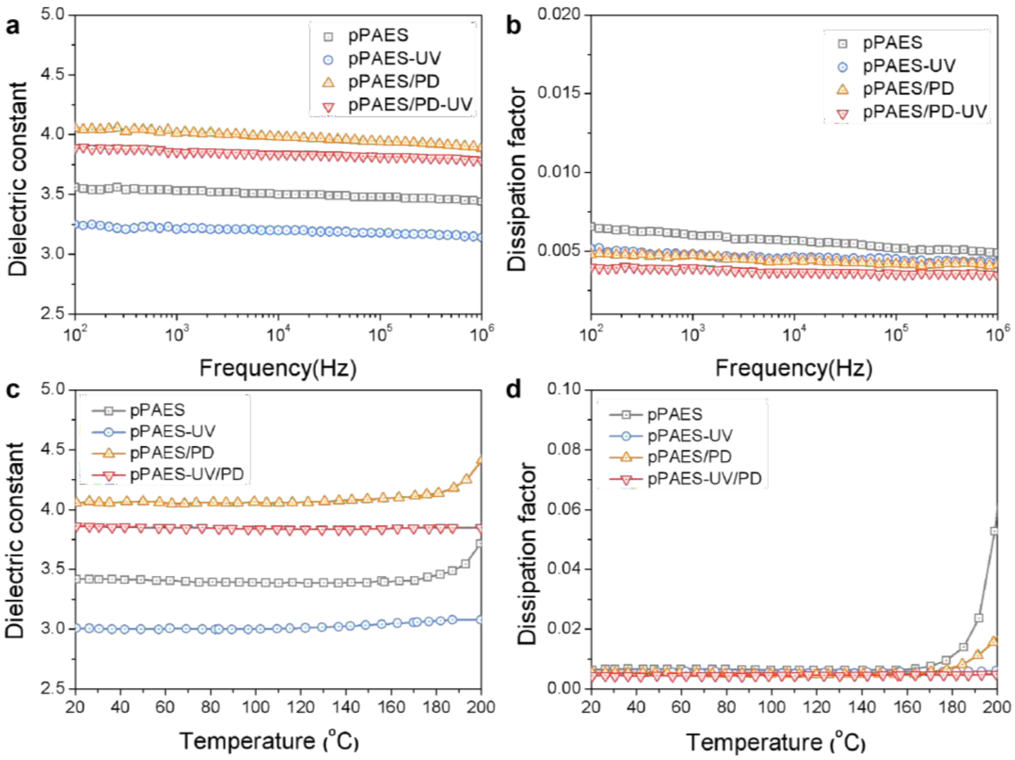

Fig. S14 Frequency-dependent (**a**) dielectric constant and (**b**) dissipation factor of pPAES, pPAES- UV, pPAES/PD and pPAES/PD-UV. Temperature-dependent (**c**) dielectric constant and (**d**) dissipation factor of pPAES, pPAES-UV, pPAES/PD and pPAES/PD-UV

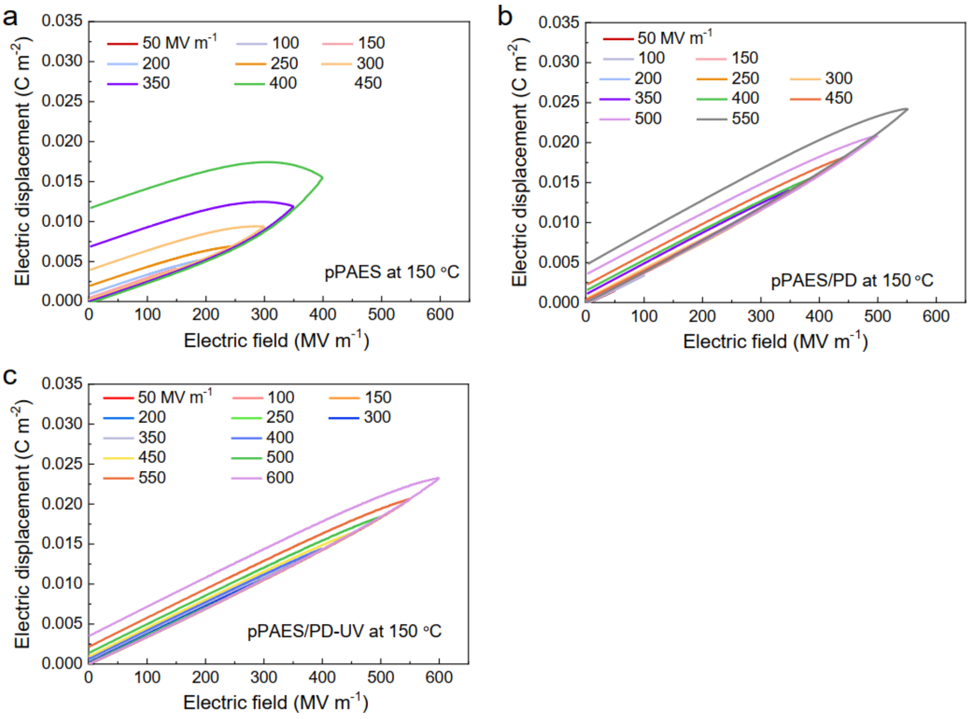

Fig. S15 Unipolar D-E loops of (**a**) pPAES, (**b**) pPAES/PD and (**c**) pPAES/PD-UV at 150 °C

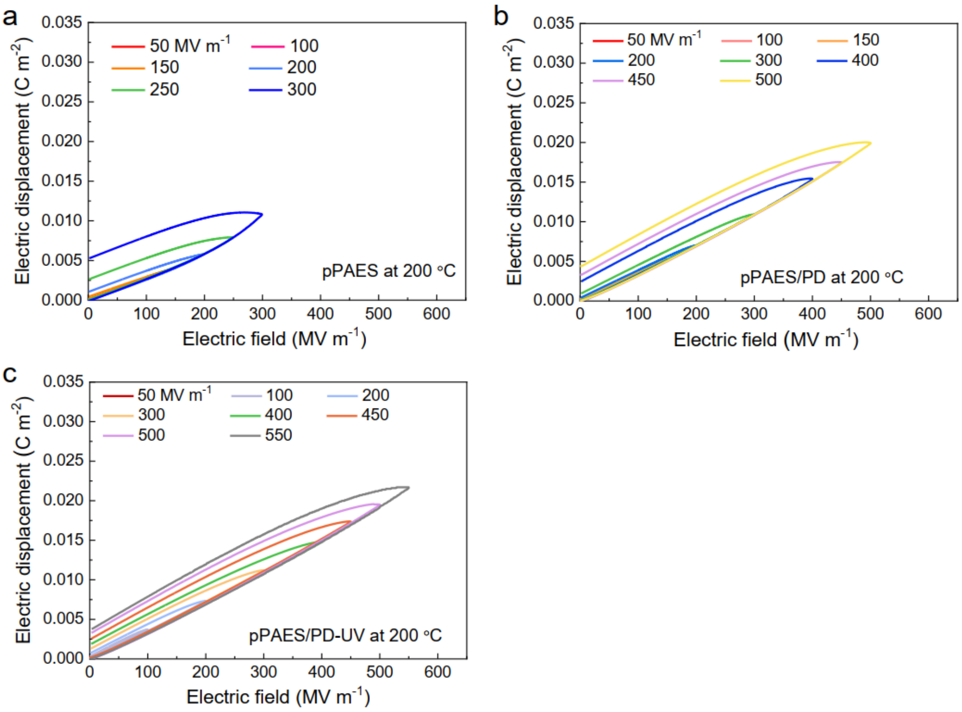

Fig. S16 Unipolar D-E loops of (**a**) pPAES, (**b**) pPAES/PD and (**c**) pPAES/PD-UV at 200 °C

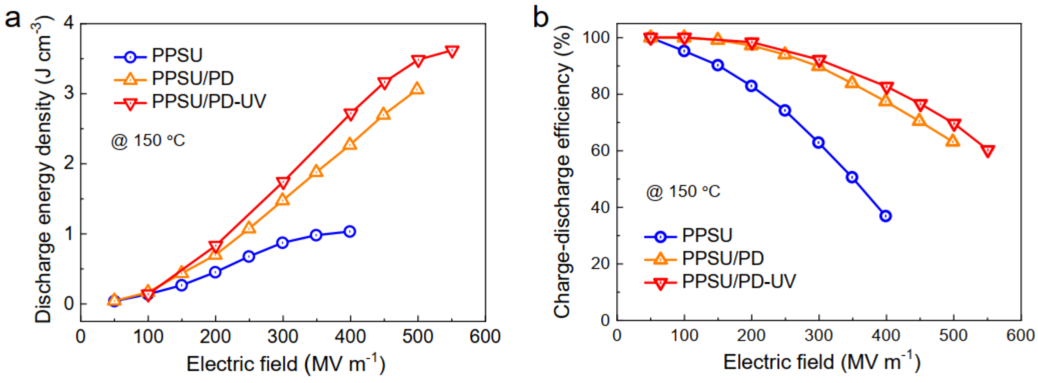

Fig. S17 (**a**) Discharged energy density and (**b**) charge-discharge efficiency of PPSU, PPSU/PD and PPSU/PD-UV at 150 °C

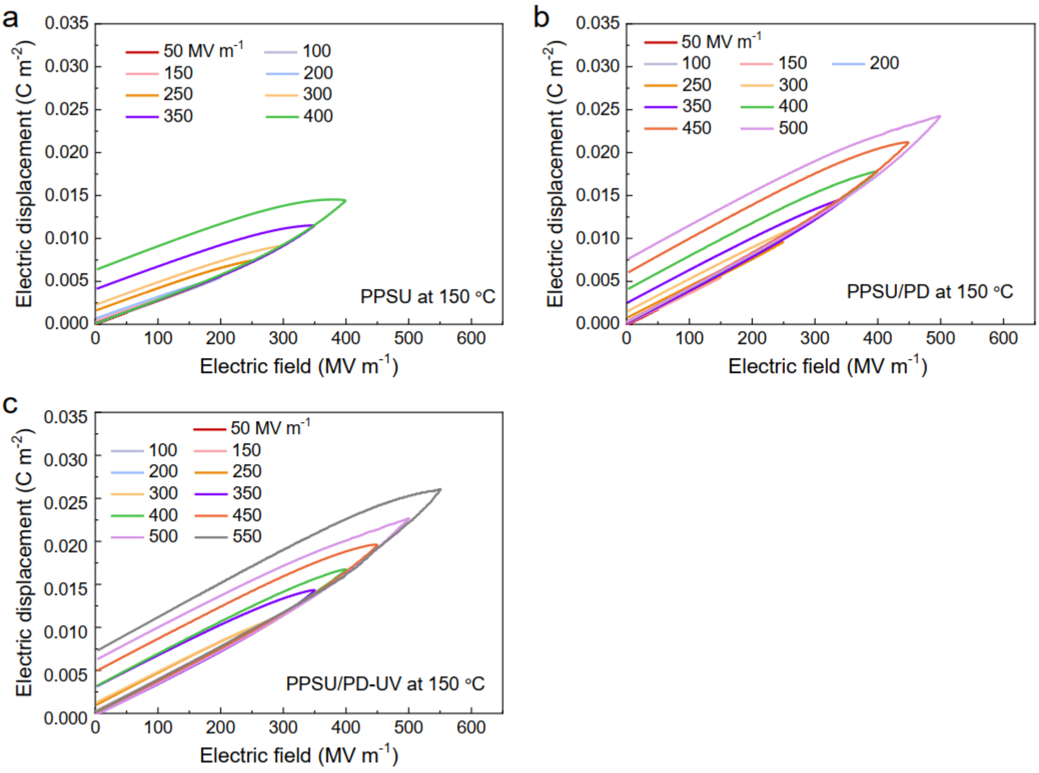

Fig. S18 Unipolar D-E loops of (**a**) PPSU, (**b**) PPSU-UV, (**c**) PPSU/PD and (**d**) PPSU/PD-UV measured at 150 °C

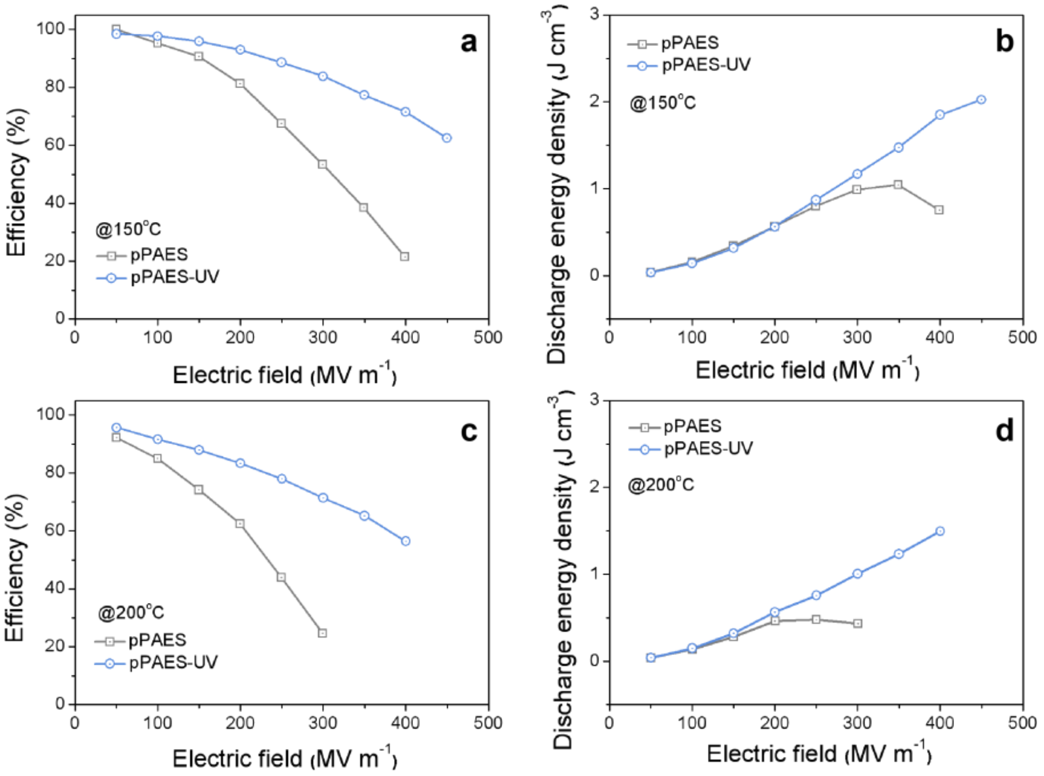

Fig. S19 Discharged energy density and charge-discharge efficiency of pPAES and pPAES-UV at 150 °C and 200 °C

The improvement in energy storage performance of pPAES/PD-UV after irradiation is the joint contribution of polymer dots and pPAES. The main text has discussed in detail the changes of composites containing polymer dots before and after UV irradiation. Here, the UV irradiation is applied to pure pPAES. As shown in Fig. S19, compared with pPAES, the values of discharged energy density (Ue) and charge-discharge efficiency (η) of pPAES-UV have been significantly improved at 150 °C and 200 °C.
